# Supplementary material for: Epithelial to Mesenchymal Transition: A Mechanism that Fuels Cancer Radio/Chemoresistance
Source: Cells. 2020 Feb 12;9(2):428. doi: 10.3390/cells9020428 (PMC7072371; doi:10.3390/cells9020428)
Supplement: Supplementary file 1 [file cells-09-00428-s001.pdf]

PRISMA 2009 Flow Diagram

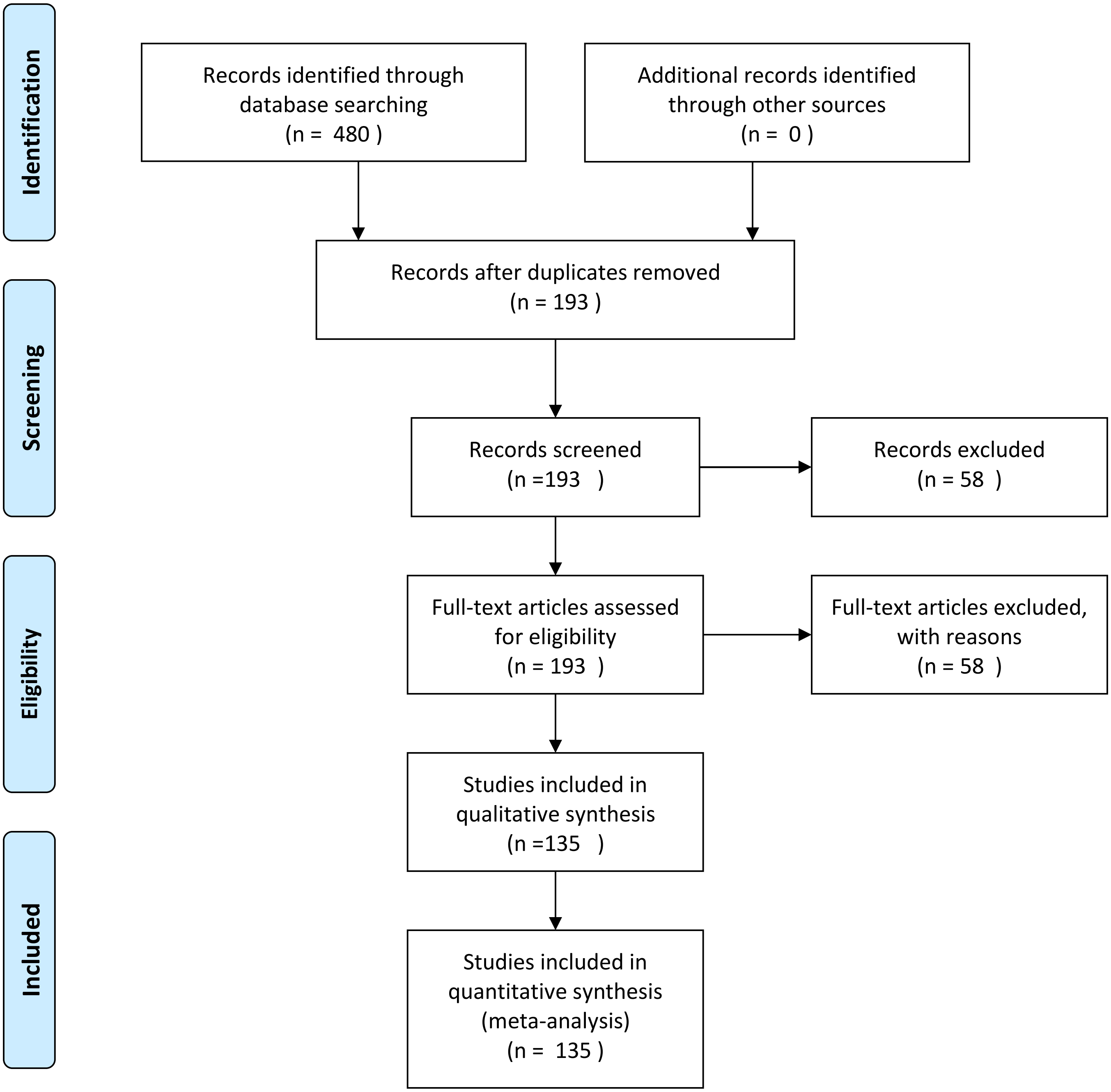

From: Moher D, Liberati A, Tetzlaff J, Altman DG, The PRISMA Group (2009). *P*referred *R*eporting *I*tems for *S*ystematic Reviews and *M*eta-*A*nalyses: The PRISMA Statement. PLoS Med 6(7): e1000097. doi:10.1371/journal.pmed1000097

For more information, visit www.prisma-statement.org.
